# Vitamin C Modulates the PI3K/AKT Pathway via Glutamate and Nitric Oxide in Developing Avian Retina Cells in Culture

**DOI:** 10.3390/brainsci15040369

**Published:** 2025-04-02

**Authors:** Aline T. Duarte-Silva, Ivan Domith, Isabele Gonçalves-da-Silva, Roberto Paes-de-Carvalho

**Affiliations:** 1 Program of Neurosciences, Institute of Biology, Fluminense Federal University, Niterói 24210-346, RJ, Brazil; aline.duarte@idor.org (A.T.D.-S.); ivan.gallo@idor.org (I.D.); isabelesilva@id.uff.br (I.G.-d.-S.); 2 Instituto D’Or de Pesquisa e Ensino (IDOR), Rio de Janeiro 22281-100, RJ, Brazil; 3 IDOR/Pioneer Science Initiative, Rio de Janeiro 22281-100, RJ, Brazil; 4 Department of Neurobiology, Institute of Biology, Fluminense Federal University, Niterói 24210-346, RJ, Brazil

**Keywords:** ascorbate, signaling pathway, calcium, NMDA and AMPA receptors, glutamate biosensor

## Abstract

**Background:** In addition to its known antioxidant function, the reduced form of vitamin C, ascorbate, also acts as a neuromodulator in the nervous system. Previous work showed a reciprocal interaction of ascorbate with glutamate in chicken embryo retinal cultures. Ascorbate modulates extracellular glutamate levels by inhibiting excitatory amino acid transporter 3 and promoting the activation of NMDA receptors and the consequent activation of intracellular signaling pathways involved in transcription and survival. **Objective:** In the present work, we investigated the regulation of AKT phosphorylation by ascorbate in chicken embryo retina cultures. **Methodology:** Cultures of chicken embryo retina cells were tested using Western blot, immunocytochemistry, fluorescent probe transfection, and cellular imaging techniques. **Results:** Our results show that ascorbate induces a concentration and time-dependent increase in AKT phosphorylation via the accumulation of extracellular glutamate, the activation of glutamate receptors, and the activation of the PI3K pathway. Ascorbate produces an increase in intracellular calcium accumulation and, accordingly, AKT phosphorylation by ascorbate is blocked by the calcium chelator BAPTA-AM. Moreover, AKT phosphorylation is also blocked by the nitric oxide synthase inhibitor 7-nitroindazole, indicating that it is mediated by calcium and nitric oxide-dependent mechanisms. **Conclusions:** We demonstrate that ascorbate modulates the PI3K/AKT pathway in retinal cultures through the activation of glutamate receptors and NO production in a calcium-dependent manner. Given that previous research has shown that glutamate induces ascorbate release in retinal cultures, our findings emphasize the significance of the reciprocal interactions between ascorbate and glutamate in retinal development. These findings provide further evidence supporting the role of ascorbate as a neuromodulator in retinal development.

## 1. Introduction

Vitamin C is found in nature as ascorbate (AA) and/or dehydroascorbate (DHA), its reduced and oxidized forms, respectively [1,2]. In its ascorbate form, vitamin C acts as an antioxidant because it can donate electrons [1]. In addition, it plays an important role in physiology as a cofactor in many enzyme reactions, including catecholamine, cholesterol, and amino acid synthesis [2].

The physicochemical characteristics of vitamin C, negatively charged in physiological pH, prevent it from crossing the lipid bilayer and therefore a transport system is necessary. Two classes of transporters for vitamin C are well known, sodium and vitamin C co-transporters (SVCTs) and glucose transporters (GLUTs), which are similar in their structures but differ in their distribution throughout the body [3]. SVCT2, mainly present in neurons, is the AA transporter expressed in nerve tissues, being distributed in the cortex, hippocampus, cerebellum, and retina [4,5]. GLUTs are responsible for the transport of DHA and also promote AA recycling by neuronal cells [6]. In the retina, some studies show that transport can be regulated by different neurotransmitters, such as dopamine, nitric oxide, and glutamate [7,8]. Interestingly, ascorbate promotes a decrease in glutamate uptake by decreasing excitatory amino acid transporters present in the membrane. As a result, it causes glutamate to accumulate in the extracellular space, which activates NMDA receptors and subsequently increases CREB phosphorylation [9].

Glutamate, the main excitatory neurotransmitter in the central nervous system, is present in about 80 to 90% of synapses in the brain [10]. Its role in the central nervous system includes several brain functions such as cognition, learning, and memory [11,12,13], synapse formation [14], differentiation and cell death [15], in addition to many others. The majority of glutamate’s effects on the nervous system are mediated through its receptors. The ionotropic class of these receptors is subdivided into NMDA, AMPA, and kainate receptors. In general, these receptors are ion channels that allow flux of cations when activated by agonists, thus activating signaling pathways [16]. Calcium ion (Ca^2+^) influx initiates the activation of various intracellular targets, including the calmodulin (CaM) pathway, which subsequently modulates several enzymes [17].

Among the enzymes associated with Ca^2+^/CaM and dependent on glutamate ionotropic receptor activities, there is the nitric oxide synthase (NOS) [18]. There are three known isoforms of NOS: neuronal, present in neurons; endothelial, predominant in endothelial cells; and inducible, expressed mainly in cells from immune system [19]. Moreover, the activation by Ca^2+^/CAM NOS activity is regulated by signaling pathways, involving PKG and PI3K [20,21].

PI3K is part of a family of enzymes capable of phosphorylating membrane phosphoinositols. For example, when receptor tyrosine kinases are activated, their conformational change allows the recruitment of PI3K, which in turn phosphorylates phosphatidylinositol generating phosphatidylinositol triphosphate (PIP3) [22]. The AKT protein has a plekstrin homologous domain that interacts with PIP3. After this interaction, AKT is recruited to the membrane and is activated when phosphorylated by mTORC2 and PDK1 in the residues ser 473 and Thr 308, respectively [23,24]. AKT is a serine threonine kinase involved in cell death [25,26], as well as the inhibition and activation of transcription factors [27,28], the regulation of mTORC1 [29], and others.

In chick retina culture, nitric oxide (NO) signaling pathways result in an increase in AKT phosphorylation in both Thr308 and Ser473, in a PI3K-dependent manner, inducing AKT translocation to the nucleus [30]. Once in the nucleus, AKT regulates the activation of transcription factors such as CREB, which in turn modulate cell survival and proliferation [31].

In the present work, we examined how vitamin C affects the PI3K/AKT signaling pathway, which regulates key intracellular processes. We show that AA regulates the PI3K/AKT pathway in retinal cultures via glutamate receptors and NO production in a calcium-dependent manner. Since previous work showed that glutamate promotes ascorbate release in retinal cultures [5], the results highlight the importance of AA/glutamate reciprocal interactions in the developing retina.

## 2. Materials and Methods

### 2.1. Animals

Fertilized White Leghorn chicken eggs were obtained from a local hatchery and incubated at 38 °C and 80–90% humidity. Procedures using chick embryos were all in accordance and registered in the local commission of animal care (CEUA) from Fluminense Federal University, under protocol 9883070622.

### 2.2. Materials

HEPES, NG-nitroL-arginine methyl ester, 6,7-dinitroquinoxaline-2,3-dione (DNQX), dimethyl sulfoxide, diamidino-2-phenylindole, dizocilpine maleate (MK-801), 40,6-diamidino-2-phenylindole, 1,2-bis-(o-Aminophenoxy)-ethane-N,N,-N′,N′-tetraacetic acid tetraacetoxymethyl ester (BAPTA-AM), wortmannin, naphthol AS-E phosphate, glycergel (DAKO), L-glutamic acid, and L-ascorbic acid were purchased from Sigma Aldrich (St Louis, MO, USA). 7- Nitroindazole (7NI) and LY 294002 were purchased from Biomol (Plymouth Meeting, PA, USA). Fetal bovine serum, trypsin, and minimum essential medium (MEM) were purchased from Gibco (Grand Island, NY, USA). Enhanced chemiluminescence kit and polyvinylidene difluoride membranes were purchased from GE Healthcare (Buckinghanshire, UK). Primary antibodies against AKT and pSer473 AKT were from Cell Signaling (Beverly, MA, USA). Alexa488 and Alexa568 conjugated secondary anti-rabbit and anti-mouse antibodies were from Molecular Probes (Eugene, OR, USA). Penicillin, streptomycin, L-glutamine, and horseradish peroxidase conjugated secondary anti-rabbit antibodies were purchased from Invitrogen (Carlsbad, CA, USA). The monoclonal antibody 2M6 for chick retinal Muller cells [32] was kindly provided by Dr. B. Schlosshauer (Max-Planck-Institute, Tubingen, Germany).

### 2.3. Retinal Culture

Retinas from eight-day-old (E8) chick embryos were dissected in calcium and magnesium-free Hank’s-balanced salt solution (CMF) and digested with 0.2% trypsin for 20 min at 37 °C. The retinal tissue was washed and mechanically dissociated with a Pasteur glass pipette in MEM supplemented with 3% fetal bovine serum, glutamine (2 mM), penicillin (100 U/mL), and streptomycin (100 µg/mL). After that, the cells were suspended in MEM, seeded in 12 well culture plates, and maintained at 37 °C in an incubator with 95% air and 5% CO_2_. Culture medium was changed for fresh medium after one day (C1) in culture and the experiments were performed on the third (C3) or fourth day (C4) of culture.

### 2.4. Treatment

Cultures grown in 12-well plates were kept serum-starved in Hank’s balanced salt solution (HBSS) for one hour and then washed and incubated with Hank’s at 37 °C. Treatments with pharmacological inhibitors were performed ten minutes before stimulation with AA. Cultures were stimulated with different concentrations of AA for different times, then lysed and collected for protein measurement.

### 2.5. Western Blotting

The cultures were washed twice with HBSS and starved for one hour. After treatments, the cultures were lysed, and the total protein amount was estimated by Bradford’s method. Samples containing 30 μg protein were submitted to 9% SDS-PAGE and proteins transferred to PVDF membranes. The membranes were blocked and incubated overnight with antibodies against phospho ser473 AKT. Subsequently, the membranes were washed in TBS-T buffer, incubated with HRP-conjugated secondary antibody, and developed using an ECL kit (Amersham, Marlborough, MA, USA). After protein stripping with 0.2 M glycine, pH 2.2, for 30 min, the membranes were re-probed with antibody against total AKT. Images were acquired in a ChemiDoc^TM^ XRS+ System (Bio-Rad Laboratories, Hercules, CA, USA) and quantified using the ImageJ Fiji software (version 1.44p) (Laboratory for Optical and Computational Instrumentation, Madison, WI, USA).

### 2.6. Cell Transfection

The calcium phosphate transfection protocol used was based on the work of Sun and colleagues [33]. Briefly, 2 μg of DNA, 12.5 µL of 2 M CaCl_2_, 20 µL of glycerol, and sterile ultrapure H_2_O for a total volume of 100 µL were added to a sterile tube. In a second sterile tube, 100 µL of HBSS was added. Next, 12.5 µL of HBSS was added 8× to the tube containing the CaCl_2_/glycerol/DNA mixture, gently vortexing for 1 s each time. The solution in the tube rested for 15 min. Then, 100 µL of the transfection solution was dripped into a well in the 12-well plate containing cultures obtained from 8-day-old embryos cultured for 1 day (E8C1) in 18 mm coverslips for 5 h. After incubation, the cells were washed 3× with HBSS pH 7.3 and a new medium was added to the well until the day of the experiment.

### 2.7. Extracellular Glutamate and Calcium Imaging

For extracellular glutamate and calcium imaging experiments, pCMV (MinDis) was used. iGLUSNFR, a glutamate biosensor, and PGP-CMV-GCAMP6F, a calcium sensor, were used. The cells were recorded for 1 min before being stimulated with 250 µM glutamate or 1.5 mM AA. AA and TBOA were pre-incubated for 20 min before glutamate stimulation. Confocal microscope (Leica TCS SP5 II) was configured in live data mode, resonant scanner on, zoom factor 3, image acquisition every 1 s, and a 63× objective. An argon laser set to 20% power was used with excitation at 488 nm and PMT1 between 500 and 550 nm. All experiments occurred at 37 °C. The first minute of imaging used as cell basal fluorescence represented by F_0._ ΔF/F_0_ provides a quantitative measure of how fluorescence changes relative to baseline. Each cell was treated as an independent unit (n), with data obtained from a minimum of 6 cells derived from at least three different cultures. Data were quantified using the LAS AF 2.0 software (Leica Microsystems).

### 2.8. Immunocytochemistry

After treatment, E8C3 cultures were fixed with 4% paraformaldehyde (PFA) in 0.16 M Phosphate buffer, pH 7.2, for 1h, washed three times for 10 min with sodium phosphate-buffered saline (PBS), and incubated for 2 h with blocking solution (5% bovine serum albumin, 5% fetal bovine serum, and 0.01% Triton X-100 in PBS). Next, the cells were incubated with a primary antibody (pSer473 AKT 1:300 and 2M6 1:200) and the slides were maintained overnight in a humidified chamber. The next day, the coverslips were washed with PBS three times for 10 min and secondary antibodies (anti-rabbit Alexa 488 1:300 and anti-mouse Alexa 568), were added and maintained for 2 h. After that, the coverslips were washed three times for 10 min with PBS. DAPI (1:1000), a nuclear marker, was added for 30 s and the cells were washed three times for 5 min. Next, the coverslips were mounted using DAKO mounting medium. The images were obtained using the Leica TCS SP5 II confocal microscope.

### 2.9. Statistical Analysis

The graphs were made using the Graphpad prism software (version 8.0.2). Statistical analyses were performed using the one-way ANOVA test followed by the Bonferroni post-test.

## 3. Results

### 3.1. AA Promotes Accumulation of Extracellular Glutamate

**Figure 1:**
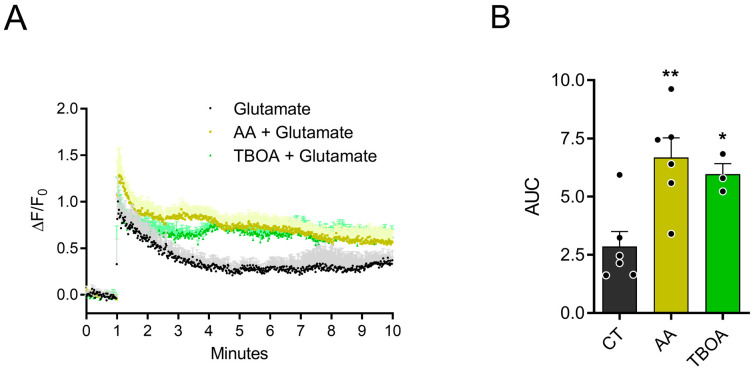
AA promotes accumulation of extracellular glutamate. (**A**) E8C3 cultures were transfected in C1 with pCMV (MinDis). iGLUSNFR, glutamate biosensor, and experiments were performed in C3. For 1 min, cells were stimulated with 250 µM glutamate. AA (1.5 mM) and TBOA (100 µM) were pre-incubated for 20 min before glutamate stimulation. ΔF/F_0_ represents relative variation in fluorescence. “F_0_” is basal fluorescence in first minute while “F” indicates fluorescence recorded throughout experiment. (**B**) Area under the curve from 1 to 10 min was calculated for each condition. (AUC CT: 2.8 ± 0.6, n = 6; AA: 6.6 ± 0.8, n = 6; TBOA: 5.9 ± 0.4, n = 3). Each value represents mean ± SEM of at least 3–6 independent cultures. * *p* < 0.05, ** *p* < 0.01 in relation to control (CT).

Previous data showed that glutamate is able to promote AA release by reversing the SVCT2 transporter in chick retina cell cultures [5]. Interestingly, AA inhibits glutamate transport, promoting an increase in extracellular glutamate, and then regulating CREB phosphorylation via the NMDA receptor [9]. Given that previous studies have shown that glutamatergic receptors activate the PI3K/AKT pathway in retinal cultures [30], we aimed to investigate if ascorbate also regulates AKT phosphorylation levels. First, in line with our previous data, we decided to conduct experiments to investigate the accumulation of extracellular glutamate over time. The cultures were transfected with pCMV (MinDis).iGLUSNFR, a glutamate biosensor, in culture day 1 (C1), and the experiments were performed in C3/C4. As can be seen in Figure 1A, after recording one minute of cell activity, the addition of 250 µM glutamate to the culture promoted an increase in the detection of glutamate by the sensor. This was followed by a decay of glutamate detection and a plateau was attained at 60/70% of maximum peak (black circles). Previous data from the group showed that 1.5 mM of AA over 30 min leads to changes in cellular signaling. In this study, treatment with 1.5 mM AA promoted a delay of glutamate decrease when compared to the control condition (yellow triangles). As expected, treatment with 100 µM TBOA, a non-selective inhibitor of excitatory amino acid transporters, also promoted a delay of glutamate decrease in the extracellular medium over 10 min. Figure 1B shows the calculated area under the curve (AUC) for each condition, with an evident increase for ascorbate or TBOA treatments. These data are consistent with the hypothesis that AA promotes the inhibition of glutamate transport and accumulation at the extracellular medium.

### 3.2. AA Promotes AKT Phosphorylation in a Concentration- and Time-Dependent Way

**Figure 2:**
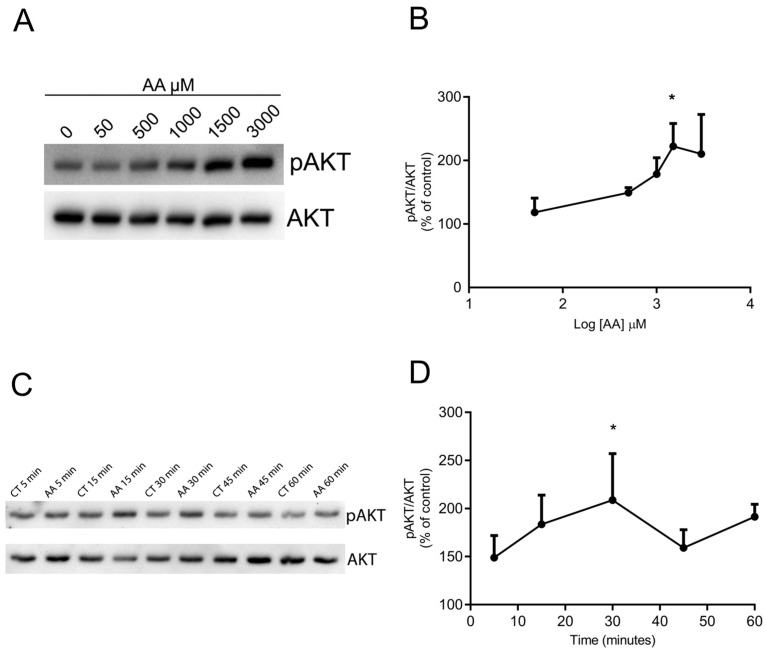
Ascorbate (AA) increases Ser473 pAKT levels in chick embryo retina culture. (**A**,**B**) E8C3 cultures were treated for 30 min with different ascorbate (AA) concentrations. (**C**,**D**) Cultures were incubated with 1.5 mM ascorbate (AA) for different times. After incubation, cells were lysed and harvested for Western blot processing using pAKT and AKT antibodies. (B: CT:100%, n = 5; AA 50 µM: 118.1 ± 22.58, n = 5; AA 500 µM: 149.0 ± 8.2, n = 4; AA 1000 µM: 178.3 ± 25.7, n = 5; AA 1500 µM: 222.1 ± 35.9, n = 4; AA 3000 µM: 210.0 ± 65.3, n = 4; D: CT 5 min: 100%, n = 4; AA 5 min: 148.9 ± 23.0, n = 3; CT 15 min: 100%, n = 4; AA 15 min: 152.1 ± 41.3, n = 3; CT 30 min: 100%, n = 4; AA 30 min: 231.4 ± 55.1, n = 4; CT 45 min: 100%, n = 4; AA 45 min: 159.1 ± 18.8, n = 3; CT 60 min: 100%, n = 4; AA 60 min: 143.7 ± 40.0, n = 4.) Each point represents mean ± SEM of 3 to 5 independent cultures. * *p* < 0.05 in relation to control.

To investigate whether AKT phosphorylation could be induced by AA treatment, the cells were treated with different concentrations of AA for different times and processed for Western blot. Figure 2A,B and Figure S1 show that incubation with ascorbate for 30 min promotes AKT phosphorylation at ser 473 in a concentration-dependent manner, beginning with 500 µM and attaining a maximum with 1.5 or 3.0 mM. Figure 2C,D shows that stimulation is also time-dependent. There was an increase in AKT phosphorylation stimulated with 1.5 mM AA observed after 5 and 15 min, attaining a maximum in 30 min, and decreasing in 45 or 60 min. These data clearly indicate that AA promotes maximum AKT phosphorylation in a concentration of 1.5 mM and within 30 min of incubation. This concentration and time were then used in the experiments thereafter.

### 3.3. AA Induces Increased pAKT in Glial and Neuronal Cells

**Figure 3:**
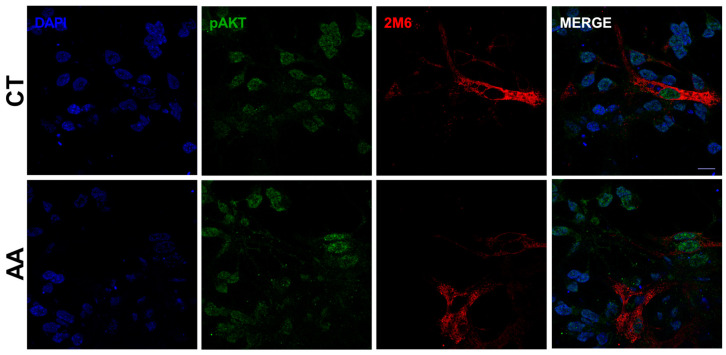
AA increases pAKT levels in glial and neuronal cells. E8C3 cultures were treated with AA 1.5 mM for 30 min. Cells were then fixed in PFA 4% and immunocytochemistry was performed. DAPI: nuclear marker; 2M6: glial antibody (1:200); pAKT: ser 473 phosphorylated AKT antibody (1:300). Notice increase in pAKT (green labeling) when cultures were exposed to AA. Presence of pAKT labeling can also be observed in nuclei (blue) mainly in 2M6-labeled Muller cells (in red).

Chicken embryo retinal cultures present neuronal cells such as photoreceptors and amacrine cells, and a specific type of glial cell, called the Müller cell. Therefore, we investigated whether the increase in pAKT levels was occurring specifically in glia or neuronal cells. The cultures were treated with 1.5 mM AA for 30 min and then fixed with paraformaldehyde 4%. To specifically label glial cells, antibodies against the Müller cell marker 2M6 were used. As seen in Figure 3 and Figure S5, AA increases AKT phosphorylation at ser 473 in both neuronal and glial cells.

### 3.4. Activation of Ionotropic Glutamate Receptors Participate in AKT Phosphorylation Induced by AA

**Figure 4:**
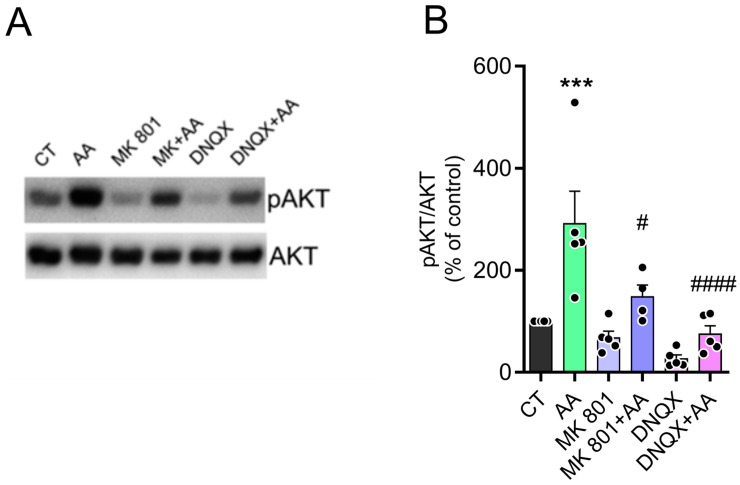
AA induces phosphorylation of AKT via NMDA and AMPA receptors. (**A**,**B**) E8C3 cultures were pretreated with 10 µM of MK801 or 200 µM of DNQX for 10 min and then treated or not with AA 1.5 mM for 30 min. Cells were lysed and harvested for Western blot processing using Ser473 pAKT and AKT antibodies. (CT: 100%, n = 4; AA: 291.3 ± 63.5, n = 5; MK801: 67.8 ± 13.0, n = 5; MK801 + AA: 148.1 ± 23.1, n = 4; DNQX: 26.6 ± 7.4, n = 5; DNQX + AA: 74.9 ± 16.1, n = 5.) Each bar represents mean ± SEM of 4–5 independent cultures. *** *p* ≤ 0.001 in relation to control, # *p* ≤ 0.05, #### *p* ≤ 0.0001 in relation to AA-stimulated.

In previous work by our group, we showed that ascorbate inhibited glutamate uptake by decreasing excitatory amino acid transporter 3 (EAAT3) in the surface of neurons. As a result of the extracellular glutamate accumulation, ascorbate is then able to activate glutamate receptors such as NMDA or AMPA receptors and activate downstream signaling pathways and promote increased pCREB levels [9]. We have now clearly demonstrated the accumulation of extracellular glutamate induced by AA using a specific glutamate sensor (see Figure 1). To explore the role of ionotropic glutamate receptors in the AA effect on AKT phosphorylation, we tested MK801 (NMDA receptor antagonist) and DNQX (AMPA receptor antagonist). The cultures were pretreated for 10 min with the antagonists and then treated with 1.5 mM AA for 30 min. As observed in Figure 4 and Figure S2, pretreatment of cultures with the antagonists MK801 or DNQX prevented AA-induced AKT phosphorylation, and these compounds had no effect alone. These findings indicate that ionotropic glutamate receptors contribute to the elevated pAKT levels induced by AA.

### 3.5. The Effect of AA on pAKT Levels Is Dependent on Calcium and Nitric Oxide (NO)

**Figure 5:**
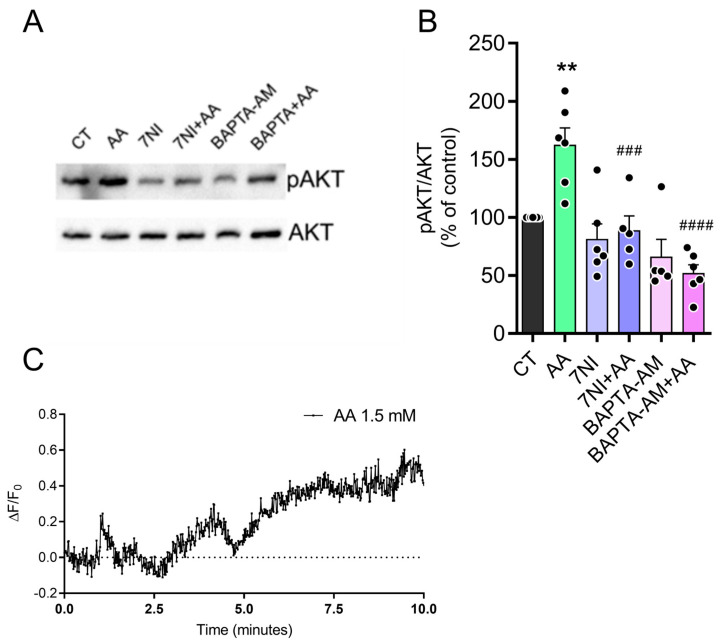
AA induces increased pAKT via calcium and nitric oxide. (**A**,**B**) E8C3 cultures were pretreated with 100 µM 7NI or 100 µM BAPTA-AM for 10 min, then cultures treated with 1.5 mM AA for 30 min. Cells were lysed and harvested for Western blot processing using ser 473 pAKT and AKT antibodies. (**C**) E8C3 cultures were transfected in C1 with PGP-CMV-GCAMP6F, a calcium biosensor, and experiments were performed in C3. In 1 min, cells were stimulated with 1.5 mM AA. ΔF/F_0_ represents relative variation in fluorescence. “F_0_” is basal fluorescence in first minute while “F” indicates fluorescence recorded throughout experiment. (B: CT: 100%, n = 7; AA: 162.3 ± 14.8, n = 6; 7NI: 80.9 ± 13.4, n = 6; 7NI + AA: 88.6 ± 12.5, n = 5; BAPTA-AM: 65.8 ± 15.2, n = 5; BAPTA-AM + AA: 51.6 ± 7.5, n = 6; C: n = 2.) Each bar represents mean ± SEM of 5–6 independent cultures. ** *p* ≤ 0.05 in relation to control, ### *p* ≤ 0.001, #### *p* ≤ 0.0001 in relation to AA-stimulated.

Calcium is an important intracellular messenger controlling essential signaling pathways for neuronal functions through biochemical cascades. It binds to calmodulin protein forming the calcium/calmodulin complex [34]. This complex activates many enzymes, including NOS, responsible for producing intracellular NO [35]. NO is an important cellular messenger that acts on several biological systems, activating soluble guanylyl cyclase to produce cyclic GMP and stimulate protein kinase G. This kinase phosphorylates targets such as AKT, ERK, among others. Data from our group have previously shown that the NO donor SNAP or L-arginine, a NOS substrate, activates signaling pathways that increase pAKT levels in chicken retina cultures [30]. To investigate whether calcium participates in the signaling pathway involved in the AA-induced increase in AKT phosphorylation, we used BAPTA-AM, a calcium chelator, for 10 min before treatment with ascorbate 1.5 mM for 30 min. As seen in Figure 5 and Figure S3, BAPTA-AM completely blocked the effect of AA. Furthermore, we used 100 μM of 7-nitroindazole (7NI), a NOS inhibitor, to evaluate the participation of NO in AKT phosphorylation promoted by AA. Figure 4 also shows that the pre-incubation of cultures with 7NI completely blocked the increase in AKT phosphorylation induced by AA, indicating the participation of NO in this effect. As shown in Figure 5C the cells were transfected with the intracellular calcium sensor PGP-CMV-GCAMP6F. After one minute of recording intracellular calcium levels, 1.5 mM AA was added to the culture. The treatment resulted in an observed increase in intracellular calcium levels over time, which is consistent with our hypothesis. Overall, these findings indicate that ascorbate treatment activates glutamate ionotropic receptors, facilitating calcium influx and activating NOS to enhance AKT phosphorylation.

### 3.6. AA Induced AKT Phosphorylation via PI3K

**Figure 6:**
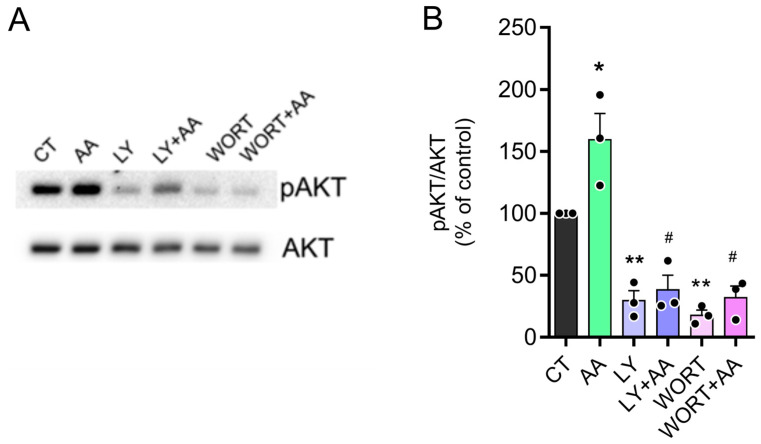
AA induces increased pAKT Via PI3K. (**A**,**B**) E8C3 cultures were pretreated with 20 µM LY294002 or 10 µM Wortmannin for 10 min and then treated with 1.5 mM AA for 30 min. Cells were lysed and harvested for Western blot processing using pAKT and AKT antibodies. (CT: 100%, n = 3; AA: 159.5 ± 21.0, n = 3; LY: 29.6 ± 7.9, n = 3; LY + AA: 38.3 ± 11.7, n = 3; WORT: 17.8 ± 4.1; n = 3; 7NI + AA: 32.1 ± 9.1, n = 3.) Each bar represents mean ± SEM of 3 independent cultures. * *p* ≤ 0.05, ** *p* ≤ 0.01, in relation to control. # *p* ≤ 0.0001, in relation to AA-stimulated.

The canonical pathway for AKT phosphorylation involves PI3K activation with the subsequent phosphorylation of phosphatidylinositol 4,5-bisphosphate to phosphatidylinositol 3,4,5-trisphosphate [36]. To evaluate the role of PI3K, we pretreated cultures with two PI3K inhibitors, LY294002 or Wortmannin, 10 min before treatment with AA 1.5 mM. Figure 6 and Figure S4 shows that both inhibitors abrogated the increase in AKT phosphorylation levels induced by AA. Interestingly, both compounds produced a decrease in pAKT control levels, suggesting a high level of PI3K activity in the cultures.

## 4. Discussion

Vitamin C exists in aqueous solution and physiological pH as AA and DHA. When absorbed by cells through sodium-dependent vitamin C transporter type 2 (SVCT2), AA reaches high concentrations in the brain (1 to 10 mM). AA is associated with many neurobiological functions such as the formation of the myelin sheath and modulation of dopaminergic and glutamatergic systems [37]. Moreover, recent studies demonstrated that AA inhibits noradrenaline transport in mouse astrocytes [38] and serotonin transport in neuroblastoma cells [39], potentially altering neurotransmitter levels in the extracellular environment and modulating intracellular signaling pathways. These findings suggest that AA interacts with multiple neurotransmitter systems, possibly influencing synaptic transmission and neuromodulation.

The retina, a part of the central nervous system, is widely modeled to study neurochemical properties of neurotransmitters, neuromodulators, and signaling pathways during embryonic development, especially in chickens [40,41]. One of the advantages of the model is the possibility to dissociate the cells and keep them in culture where they develop many properties of intact tissue [42,43]. However, important anatomical and physiological differences must be considered when translating findings to mammalian systems. The chicken retina is avascular, whereas mammalian retinas are typically vascularized [40]. Additionally, Müller glia in birds exhibit greater regenerative capacity than in mammals, potentially supporting neuroprotective responses [44].

High levels of AA are found in the developing chicken retina, but these levels decrease considerably after the hatching period [45].

Our previous data show that AA modulates the glutamatergic system in chicken retina cultures by inhibiting glutamate uptake, thus promoting the consequent activation of glutamate ionotropic receptors and calcium-dependent signaling pathways, leading to phosphorylation of the transcription factor CREB [9]. Interestingly, glutamate promotes AA release from retinal cells [8,46]. We have previously published work demonstrating that chlorogenic acids (CGAs), including caffeic acid (CFA), but also antioxidants, inhibit GDH activity in a dose-dependent manner, with complete inhibition at high concentrations [47]. This finding reinforces the hypothesis that antioxidant compounds, such as AA, may also modulate the activity of GDH or other enzymes associated with glutamate metabolism, especially considering the intracellular redox environment. GDH is an enzyme highly regulated by several ligands, including nucleotides, amino acids, zinc, and other molecules. Therefore, it is plausible that AA, due to its antioxidant properties and ability to modulate the redox environment, may indirectly influence GDH activity and, consequently, glutamate metabolism.

Since we have previously shown that glutamate promotes AKT phosphorylation in retinal cultures [30], we decided to study whether AA would be able to regulate the phosphorylation of AKT, a protein involved in many cellular functions such as survival, proliferation, and metabolism.

First, we demonstrated with a glutamate sensor that AA treatment reduced glutamate uptake, leading to increased extracellular levels compared to the control. Interestingly, a similar effect was observed with TBOA, a potent excitatory amino acid transporter blocker, demonstrating that the glutamate sensor is sensitive in detecting extracellular glutamate levels. Thus, this finding confirms previous data from our group showing an increase in glutamate extracellular levels after treatment with AA [9].

In the present study, we used mixed retinal cultures containing neuronal and glial cells. AA produced a concentration- and time-dependent increase in Ser473 AKT phosphorylation in the cultures with a maximum of approximately 150% stimulation observed with 1.5 mM in 30 min of incubation. The mechanisms that may contribute to the decrease in AKT phosphorylation after 30 min of AA treatment likely involve factors such as phosphatase activity, such as PTEN or PP2A, glutamate receptor desensitization, feedback inhibition, or downstream signaling modulation. To observe in which cell types the phosphorylation levels were increased, we performed immunocytochemistry after treatment with ascorbate and the results showed that phosphorylated AKT is increased in both neuronal and glial cells. Interestingly, AKT phosphorylation immunoreactivity could be detected in cell nuclei, as already observed when these cultures were stimulated with the NO donor SNAP [30].

Previous data indicate that AA increases extracellular glutamate by reducing EAAT3 transporters in the membrane, leading to NMDA receptor activation [11]. AMPA receptors are also present in the cultures modulating ERK activation and NO production [48]. Since glutamate and NO were also shown to stimulate AKT phosphorylation in the cultures [30], we investigated if these receptors were involved in the AKT phosphorylation increase induced by AA. Indeed, we have now shown here that the ionotropic glutamate receptor antagonists MK801 or DNQX, respectively, NMDA and AMPA receptor antagonists, were able to block AKT phosphorylation induced by AA. While previous studies have suggested that AA can interact with NMDA receptors, decreasing their function in cortical neurons, our findings indicate a different effect in our experimental conditions [49]. Our present data demonstrate that AA increases AKT phosphorylation in a manner dependent on NMDA and AMPA receptor activities. Given that NMDA receptors and certain configurations of AMPA receptors activate calcium influx, a key upstream event in several signaling pathways, we then explored its potential role in the effect observed. Additionally, nNOS activation is a key downstream effector of calcium signaling and plays a significant role in various cellular processes. Some studies have shown that NO regulates cell survival in purified cultures of chicken embryo retina neurons [50] and also regulates AKT phosphorylation and its translocation to the nucleus [30]. Our data show that the use of the calcium chelator BAPTA-AM and the nNOS inhibitor 7NI blocked AKT phosphorylation induced by AA, suggesting the participation of the calcium/calmodulin/nNOS pathway in this effect.

Several studies showed that activation of glutamate receptors is coupled to the production of NO in different CNS areas, including the retina [41,51,52]. Glial cells in chicken retina cultures do not express nNOS, eNOS, or iNOS neither L-citrulline, a coproduct of the rection catalyzed by NOS [7,53]. Our previous evidence also demonstrated that NO produced by neurons diffuses out and reaches glial cells in the cultures where it activates signaling pathways leading to CREB phosphorylation [54]. Accordingly, we suggest that NO produced by neuronal cells in retinal cultures stimulated by AA also diffuses out from neurons reaching glial cells and thus activating signaling pathways such as PI3k/AKT. Since phosphorylated AKT migrates to cell nucleus in the cultures [30] and is involved in CREB phosphorylation and neuroprotection as well [55], it is possible that both events are in the same pathway, that is, AA promotes extracellular glutamate increase and the stimulation of the PI3K/AKT/CREB pathway.

The activation of the canonical PI3K/AKT pathway is primarily responsible for the various actions triggered by AKT and the activation/inhibition of its intracellular targets [56]. It was shown in the literature that the administration of AA produces antidepressant behavior in rats, an effect mediated by the activation of PI3K and mTOR, the inhibition of GSK3β, and the increased expression of PSD-95 [57]. Here, we show that inhibitors of the PI3K pathway blocked the phosphorylation of AKT induced by AA. Data reported in the literature also show that intracellular calcium levels can regulate the activation of the PI3K/AKT pathway [58] and that the coupling of calcium with AA is important for regulating different molecular mechanisms [59]. Thus, our data corroborate previous findings by demonstrating that ascorbate modulates AKT phosphorylation via the PI3K pathway, with calcium playing a critical role in this regulation. This reinforces the complex interaction among calcium signaling, PI3K/AKT activation, and the neuromodulatory effects of AA, underscoring its relevance in intracellular signaling mechanisms essential for neuronal function and survival.

Finally, it is important to mention that studies in the literature have shown that the pathological processes related to neurodegenerative diseases and neuropsychiatric disorders are diminished with nutritional interventions as co-therapy [60,61,62,63,64]. The beneficial role of vitamin C in macular degeneration of the retina was also shown [65]. It is believed that the anti-inflammatory, antioxidant, and antiexcitotoxic role of AA is responsible for this protective action [65,66,67]. Many of these diseases are related to intense glutamatergic activity and a high increase in oxidative stress. Our results show that AA promotes the accumulation of glutamate in a non-excitotoxic manner [9], and this accumulation promotes neuromodulatory effects through the stimulation of signaling pathways involved in the survival, proliferation, and modulation of intracellular signaling. Unpublished data from our group show that AA can promote increased neuronal survival and dendritic arborization in purified neuronal cultures, an important mechanism for neural plasticity. Vitamin C recycling also regulates neurite outgrowth in neurospheres [68] and remielynation [69]. Taken together, these data show that vitamin C acts as a neuromodulator of the nervous system. 

Our results show significant effects of intracellular signaling modulation induced by vitamin C. However, some limitations can be attributed to the use of vitamin C. Among them are the fact that AA can be rapidly oxidized to DHA due to its instability in aqueous solutions. On the other hand, one of the destinations for DHA is its recycling to AA [70]. Studies also show promising effects in treatment with high doses of vitamin C administered parenterally; however, this form of administration is used more in scientific studies. Usually, vitamin C is administered orally and when passing through the gastrointestinal system, there is little absorption by cells [71]. Studies also show that pretreatment with vitamin C seems to be more effective than post-treatment in conditions of damage, functioning almost as a preventive therapy. In general, the concentrations of vitamin C used in vitro and in vivo models are not easily replicated for plasma concentrations present in humans [72].

## 5. Conclusions

**Figure 7:**
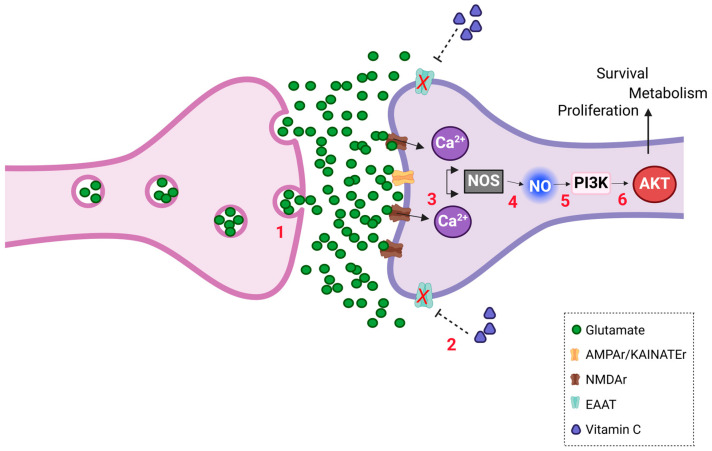
AA induces increased AKT phosphorylation. (1) Glutamate is released from presynaptic neuron into synaptic cleft. (2) Vitamin C treatment inhibits glutamate reuptake by EAAT3. (3) Glutamate accumulation increases activation of ionotropic NMDA and AMPA/KAINATE receptors in postsynaptic cell. Therefore, intracellular calcium levels are increased. Calcium interacts with calmodulin forming complex that activates enzyme NOS. (4) NOS activation induces increase in NO production. (5) NO diffuses rapidly and activates PI3K signaling pathway through unknown mechanism. (6) PI3K/AKT signaling pathway is activated, promoting changes in intracellular signaling cascades that regulate cellular processes such as survival, proliferation, and metabolism. Figure created in bioRender platform.

In summary, our results presented in Figure 7 indicate that AA promotes AKT phosphorylation through glutamatergic signaling and the activation of the PI3K pathway mediated by calcium and NO-dependent processes. These findings add evidence to support the role for AA as a neuromodulator in the developing retina.

## Data Availability

The original contributions presented in the study are included in the article. Further inquiries can be directed to the corresponding author.

## Supplementary Materials

The following supporting information can be downloaded at: https://www.mdpi.com/article/10.3390/brainsci15040369/s1. The figures related to the Western blot and immunocytochemistry experiments were included as supplementary material, ensuring a detailed and comprehensive presentation of the results. Figure S1: Original blots of Figure 2. Figure S2: Original blots of Figure 4. Figure S3: Original blots of Figure 5. Figure S4: Original blots of Figure 6. Figure S5A: Immunocytochemistry images. Figure S5B: Immunocytochemistry images.
