# Supplementary material for: Vitamin C Modulates the PI3K/AKT Pathway via Glutamate and Nitric Oxide in Developing Avian Retina Cells in Culture
Source: Brain Sci. 2025 Apr 2;15(4):369. doi: 10.3390/brainsci15040369 (PMC12025763; doi:10.3390/brainsci15040369)
Supplement: Supplementary file 1 [file brainsci-15-00369-s001.zip › brainsci-3534687-supplementary.pdf]

Supplementary material

Supplementary Figure S1: **Original blots of figure 2.**

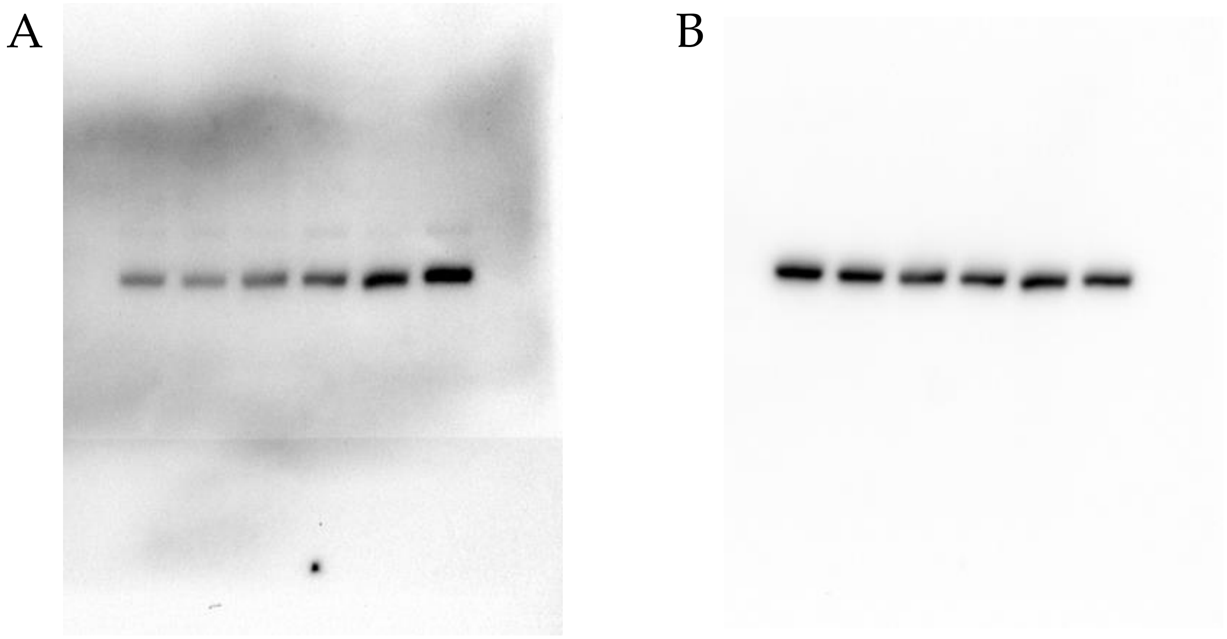

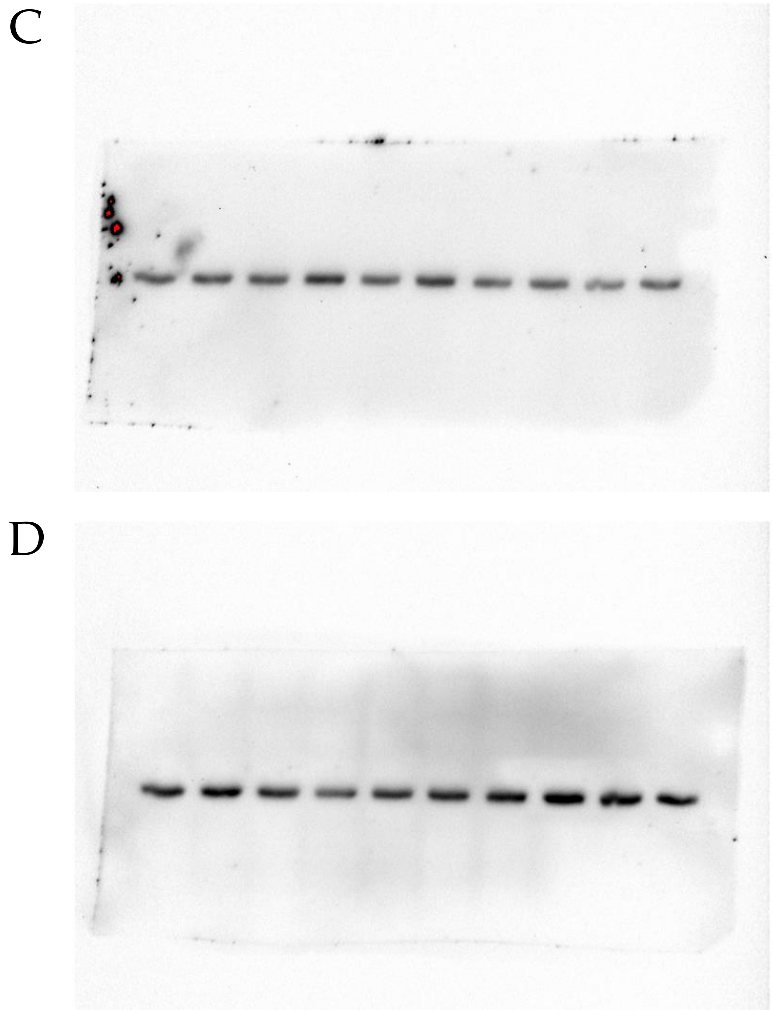

Supplementary Figure S1: Ascorbate (AA) increases Ser473 pAKT levels in chick embryo retina culture. (A and B) E8C3 cultures were treated for 30 minutes with different ascorbate concentrations. A- pSer 473 AKT and B- AKT total; Conditions: CT, AA 50 µM, AA 500 µM, AA 1000 µM, AA 1500 µM, AA 3000 µM; (C and D) Cultures were incubated with 1.5 mM ascorbate (AA) for different times. C- pSer 473 and D- AKT total; Conditions: CT 5 min, AA 5 min, CT 15 min, AA 15 min, CT 30 min, AA 30 min, CT 45 min, AA 45 min, CT 60 min, AA 60 min.

Supplementary Figure S2: **Original blots of figure 4.**

A

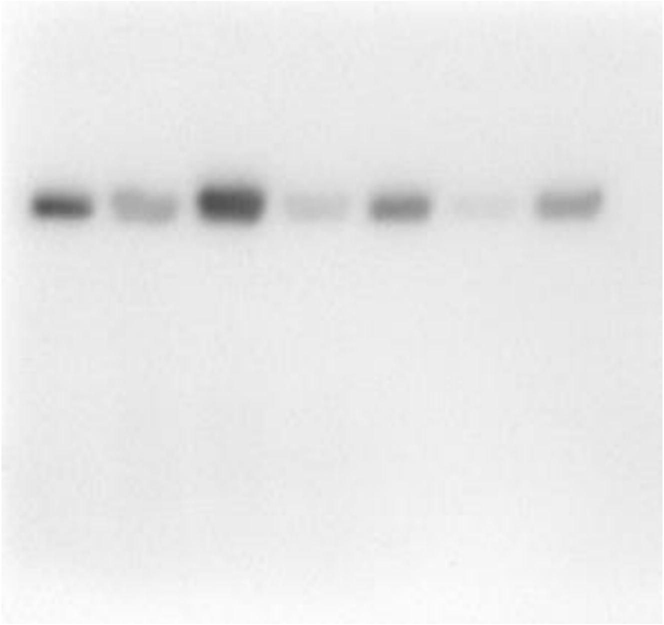

B

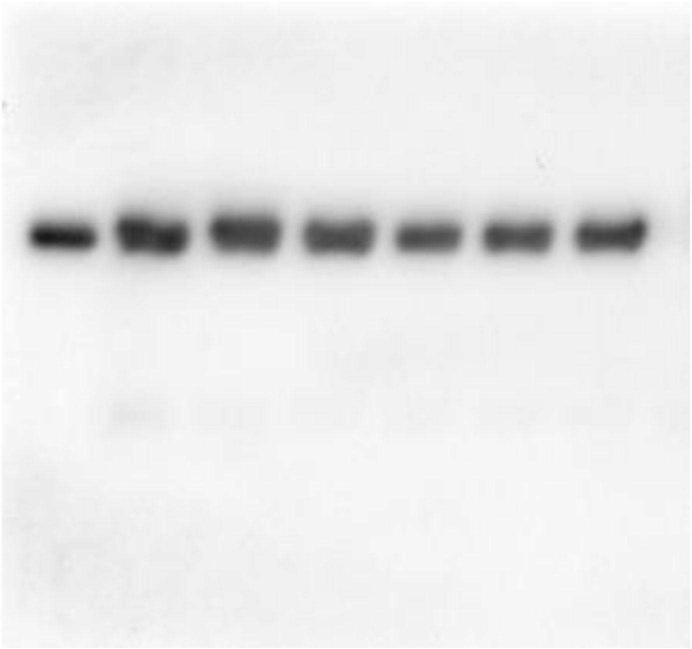

Supplementary Figure S2: AA induces the phosphorylation of AKT via NMDA and AMPA receptors. (A and B) E8C3 cultures were pretreated with 10 μM of MK801 or 200 μM of DNQX for 10 minutes and then treated or not with AA 1.5 mM for 30 minutes. A- pSer 473 AKT and B- AKT total. The first band refers to another experimental condition unrelated to this experiment. From the second band onwards, the order is: CT, AA, MK801, MK801 + AA, DNQX, DNQX + AA.

Supplementary Figure S3: **Original blots of figure 5.**

A

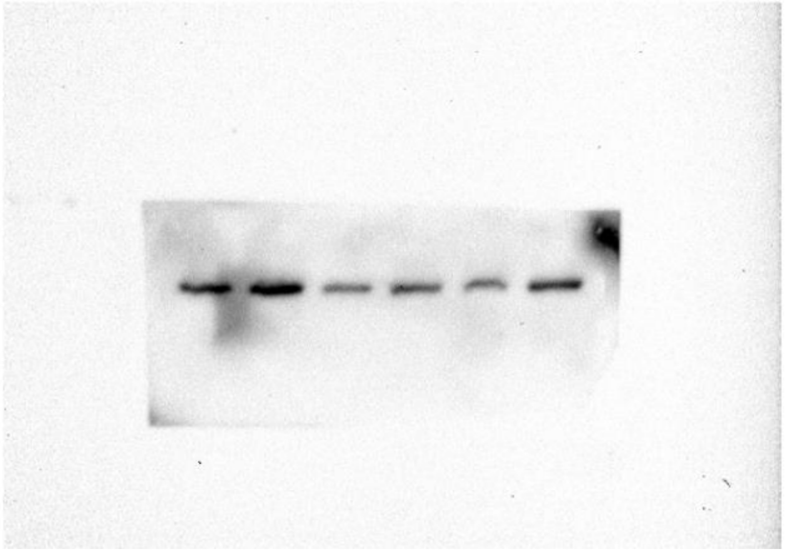

B

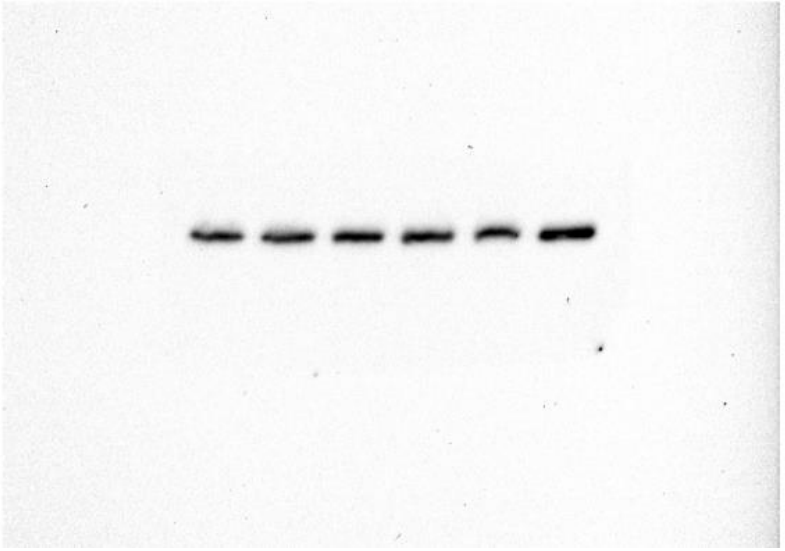

Supplementary Figure S3: AA induces increased pAKT via calcium and nitric oxide. (A and B) E8C3 cultures were pretreated with 100 µM 7NI or 100 µM BAPTA-AM for 10 minutes, then cultures treated with 1.5 mM AA for 30 minutes. A- pSer 473 AKT and B – AKT total. Conditions: CT, AA, 7NI, 7NI + AA, BAPTA-AM, BAPTA-AM+ AA.

Supplementary Figure S4: **Original blots of figure 6.**

A

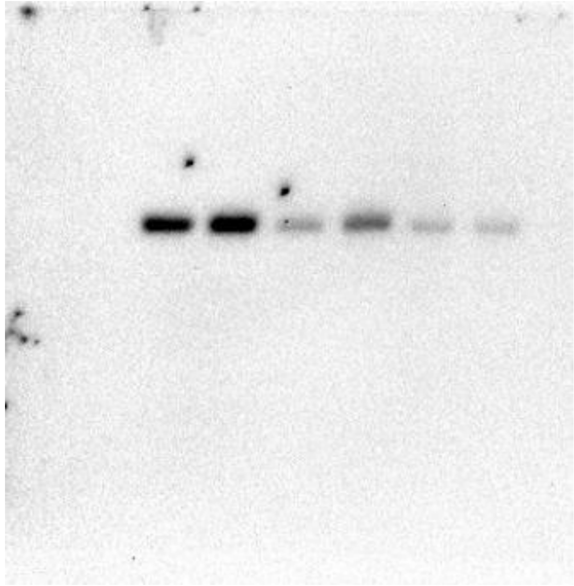

B

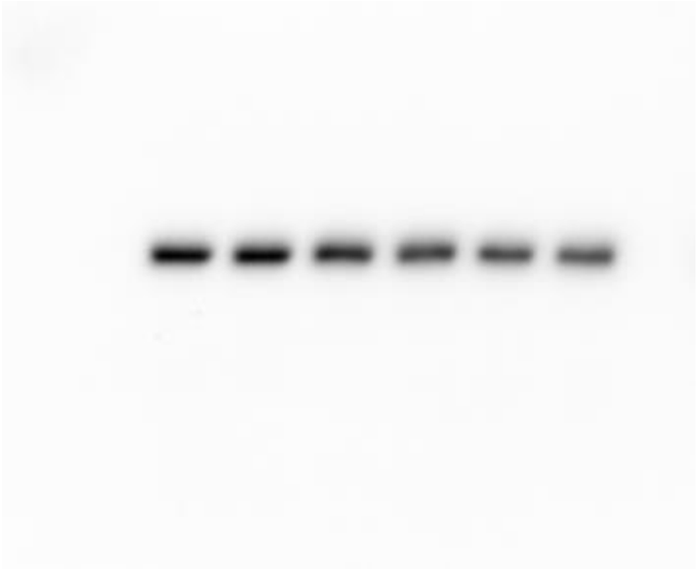

Supplementary Figure S4: AA induces increased pAKT via PI3K. (A and B) E8C3 cultures were pretreated with 20 μM LY294002 or 10 μM Wortmannin for 10 minutes and then treated with 1.5 mM AA for 30 minutes. A- pSer 473 AKT and B- AKT total. Conditions: CT, AA, LY, LY + AA, WORT, WORT +AA.

Supplementary Figure S5A: **Immunocytochemistry images**

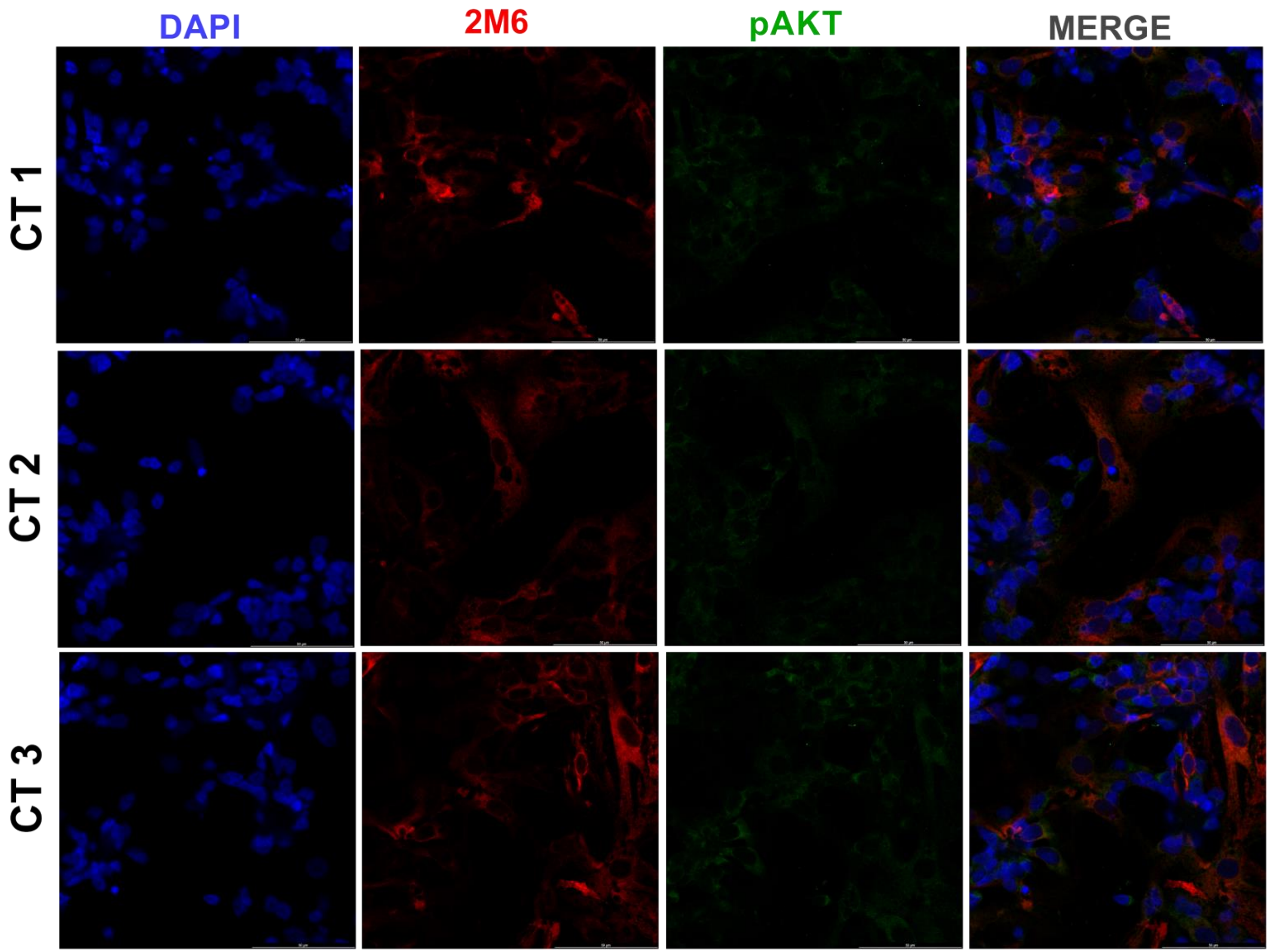

Supplementary Figure S5A: Images of three control samples for DAPI, pAKT and 2M6 staining, followed by a merged image. DAPI: nuclear marker; 2M6: glial antibody (1:200); pAKT: ser 473 phosphorylated AKT antibody (1:300).

Supplementary Figure S5B: **Immunocytochemistry images**

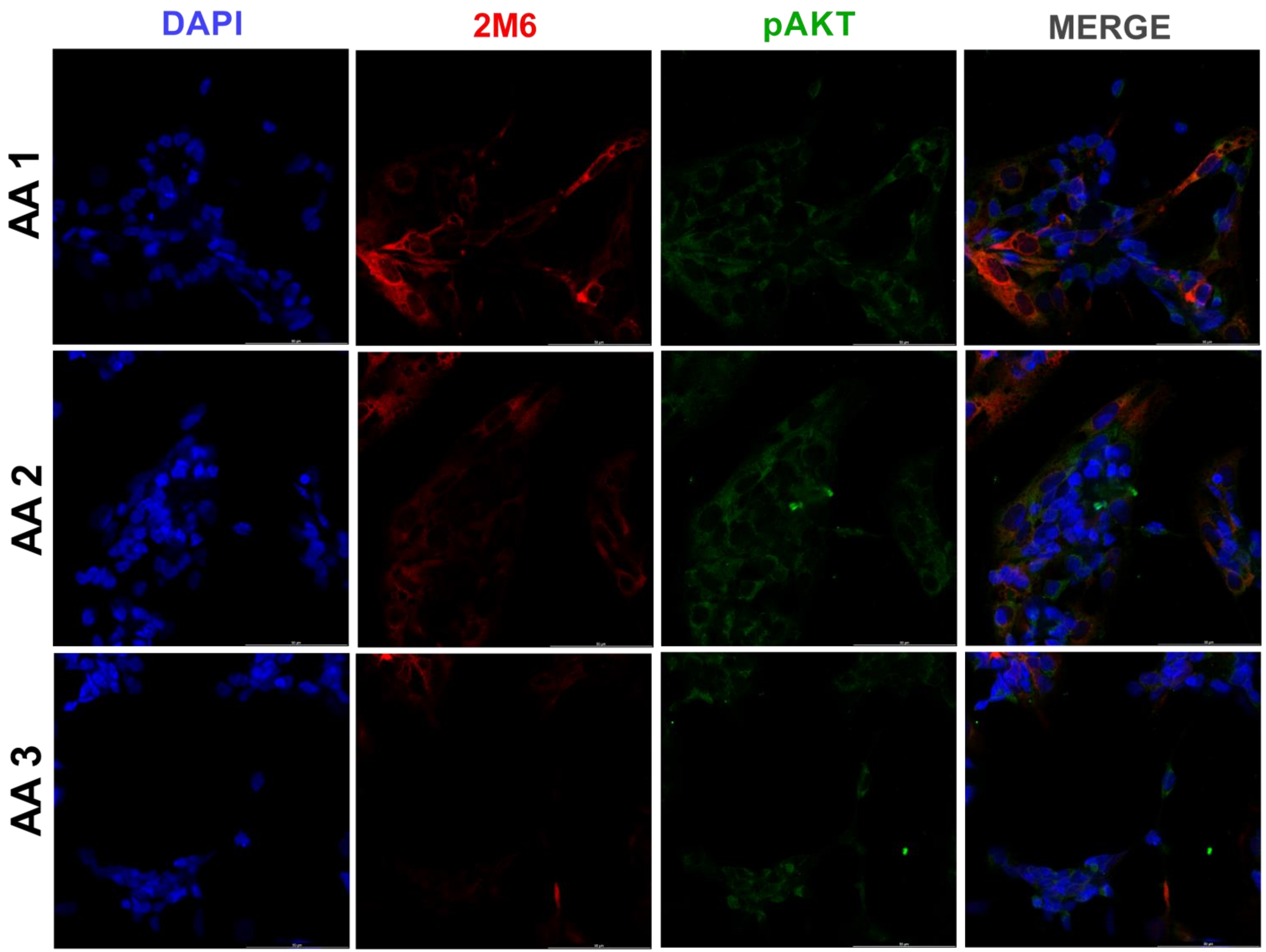

Supplementary Figure S5B: Images of three ascorbate-treated samples for DAPI, pAKT and 2M6 staining, followed by a merged image. DAPI: nuclear marker; 2M6: glial antibody (1:200); pAKT: ser 473 phosphorylated AKT antibody (1:300).
